# Does minimally invasive non-surgical therapy improve outcomes in splinted mandibular incisors? Results from a randomized trial

**DOI:** 10.1186/s12903-026-08788-4

**Published:** 2026-06-05

**Authors:** Shaimaa Hamdy, Mohammed Turky, Ahmed Y. Gamal

**Affiliations:** 1 Department of Oral Medicine, Oral Diagnosis and Periodontology, Faculty of Dentistry, Nahda University, Beni Swef, Egypt; 2 Ministry of Health and Population, Minia, Egypt; 3 Department of Endodontics, Faculty of Dentistry, Minia University, Minia, Egypt; 4 Department of Endodontics, Faculty of Dentistry, Sphinx University, Assiut, Egypt; 5 Department of Oral Medicine, Oral Diagnosis and Periodontology, Faculty of Dentistry, Ain Shams University, Cairo, Egypt; 6 Department of Oral Medicine, Oral Diagnosis and Periodontology, Faculty of Dentistry, Misr University for Science and Technology, Cairo, Egypt

**Keywords:** Periodontitis, Splinted teeth, Conventional non-surgical periodontal therapy, Minimally invasive non-surgical techniques, Intrabony defect

## Abstract

**Background:**

Conventional non-surgical periodontal therapy (CNST) is an effective method for managing mild to moderate periodontitis. However, in cases of severe periodontitis, CNST may leave residual pockets. Severe periodontitis, particularly in stages III and IV, often involves significant tooth mobility, which may require the use of a permanent splint. Minimally invasive non-surgical techniques (MINST) have been proposed to deliver better results and enhance patient satisfaction. However, current evidence does not decisively evaluate the outcomes of splinted teeth following MINST. Therefore, this research aimed to address the knowledge gap by assessing the clinical outcomes of MINST compared to CNST in the treatment of splinted teeth affected by stage III and IV periodontitis.

**Methods:**

A parallel-arm, single-blind randomized controlled trial was conducted on 39 patients (46 sites) with stage III–IV periodontitis and grade II–III mobility of mandibular incisors. Participants were randomly assigned to conventional non-surgical therapy (CNST) or minimally invasive non-surgical therapy (MINST), both of which included splinting. Outcomes were assessed at baseline, 3, and 6 months. The primary outcome was pocket reduction, assessed by changes in probing pocket depth (PPD) from baseline to follow-up. Secondary outcomes included pocket closure (PC), defined as the proportion of sites with PPD ≤ 4 mm and no bleeding on probing (BOP), derived from these measurements; clinical attachment level (CAL); defect bone fill (DBF); and patient satisfaction.

**Results:**

Both groups showed significant reductions in probing pocket depth and gains in CAL (*p* < 0.001). Pocket closure occurred more frequently in the MINST group (65.2%) compared to the CNST group (39.1%) at 6 months (*p* = 0.015). Radiographic bone fill improved significantly in both groups, with no intergroup difference. Patient satisfaction was similar (NPS: CNST = 38; MINST = 42; *p* = 0.39).

**Conclusion:**

Both MINST and CNST with splinting improved periodontal outcomes, but MINST achieved a higher rate of pocket closure, while radiographic bone fill and patient satisfaction were comparable between groups.

**Trial registration:**

Current Controlled Trials: NCT06772506.

The date of registration in the (2025–01-03) “Retrospectively registered”.

## Introduction

Periodontitis is an inflammatory multifactorial disease initiated by pathogenic bacteria accumulated as a biofilm on the tooth surface, leading to clinical attachment loss (CAL), periodontal pocket depth (PD), bleeding on probing (BOP), and bone loss, which, if neglected, may lead to increased tooth mobility and final tooth loss [1]. Pathological tooth mobility may arise from extensive alveolar bone loss, traumatic occlusion, acute periodontal inflammation, and apical tooth displacement. Treating tooth mobility can be a challenging process, particularly for patients with severe periodontitis (Stage III or IV) who have compromised periodontal support. In such cases, clinicians often consider adjunctive measures such as occlusal adjustment and tooth splinting, although the evidence for their impact on periodontal outcomes remains debated [2, 3].

Non-surgical periodontal therapy employs a closed technique to eliminate supra- and subgingival biofilm and calculus, thereby reducing periodontal inflammation and restoring periodontal health [4]. Conventional non-surgical periodontal therapy (CNST) is effective for managing mild to moderate periodontitis [5, 6]. However, it becomes challenging to eliminate subgingival biofilms in deep periodontal pockets ˃ 5 mm with either horizontal or vertical bone loss, frequently leaving residual pockets [7–9]. For more effective outcomes and patient satisfaction, periodontal non-surgical and surgical therapies are rapidly moving into minimally invasive approaches. It is hypothesized that the healing process following the minimally invasive non-surgical technique (MINST) and minimally invasive surgical procedures is similar due to the superior stability of the blood clot. This stability allows the healing process to replace the clot with a new connective tissue attachment [10–12].

To improve treatment outcomes, mobile teeth can be splinted to redistribute forces to their immobile neighboring teeth. This approach helps reduce occlusal trauma and enhances the patient's oral comfort. A consensus reached at the World Workshop in 2017 introduced a new classification for periodontal diseases, suggesting that splint treatment may be necessary for teeth exhibiting increased mobility [10]. Splinting can delay tooth extraction and the need for more complex and costly prosthodontic procedures, prolonging the lifespan of movable teeth [11].

To date, there has been no research on the effects of combined therapy, including MINST and tooth splinting, for treating patients with stage III or IV periodontitis. Therefore, this clinical study aimed to address this knowledge gap by evaluating the clinical outcomes of MINST compared to CNST in improving the clinical outcomes of permanently splinted mandibular incisors affected by stage III and IV periodontitis. We hypothesized that there is no difference between MINST + splinting and CNST + splinting in mean pocket depth reduction and in the proportion of sites achieving pocket closure (PPD ≤ 4 mm with no BOP) at 6 months.

## Materials and methods

### Study population

The current investigation was designed as a parallel-arm, single-blind, superiority randomized controlled trial (RCT) with a six-month follow-up period, including 46 sites with stage III and IV periodontitis and lower incisor teeth mobility. Each patient contributed a maximum of two teeth with intrabony defects. The participants were selected from the outpatient clinic at the Department of Oral Medicine, Oral Diagnosis, and Periodontology, Faculty of Dentistry, Nahda University. The study was conducted in alignment with the International Conference on Harmonization Good Clinical Practice Guidelines, the Declaration of Helsinki, and the Faculty of Dentistry, Minia University's research ethics registration (No. 988), committee (No. 111). This study was registered in ClinicalTrials.gov, U.S. National Library of Medicine (ID number: NCT06772506).

### Patient collection

All patients were diagnosed with stage III and IV periodontitis according to the 2017 World Workshop Classification of Periodontal Diseases [12].

Inclusion criteria:

- Patients of both sexes, aged 18 to 60 years.
- Periodontitis stage III and IV, and grade A or B.
- Selected teeth mobility ranges from grade 2 to 3.
- Single-rooted mandibular incisor teeth.
- The existence of an intrabony component ≥ 2 mm and an interdental periodontal defect with PPD ≥ 5 mm and CAL ≥ 5 mm.
- According to the Modified Cornell Index, the patients had no systemic medical conditions that may have altered their periodontal state or interfered with healing [13].
- During the past six months, no antibiotics or drugs that impact bone or soft tissue health were taken.
- Exclusion criteria:
- Multi-rooted teeth.
- Teeth with root fractures.
- Teeth with internal or external root resorption.
- Smoker patients with more than 10 cigarettes/day.
- Pregnant or nursing women.
- Patients who received regenerative periodontal therapy within six months before the initial assessment.
- Patients with a full-mouth plaque score [FMPS] of 20% who fail to practice proper oral hygiene after the first step of periodontal therapy.

### Sample size calculation

Sample size estimation was performed using G*Power 3.1 for repeated measures ANOVA (within, between interaction). Pilot data indicated a mean difference in PPD reduction corresponding to an effect size of Cohen’s f = 0.40, which was derived from a pilot dataset (*n* = 20 patients; 10 per group) conducted prior to the main study. With α = 0.05, power = 0.95, and three time points, the minimum required sample size was 24 sites (12 per group). To compensate for an anticipated 15% dropout, the target enrollment was increased to 28 sites (14 per group). Ultimately, 46 sites from 39 patients were analyzed to maintain adequate statistical power, with no loss to follow-up. Although the sample size calculation was based on a repeated-measures ANOVA framework, the final analysis employed linear mixed models and generalized estimating equations to account for clustering of multiple sites within patients. These methods are more appropriate for correlated data and typically provide equal or greater statistical efficiency; therefore, the sample size achieved is considered adequate for the applied analytical approach.

### Randomization and blinding

**Fig. 1:**
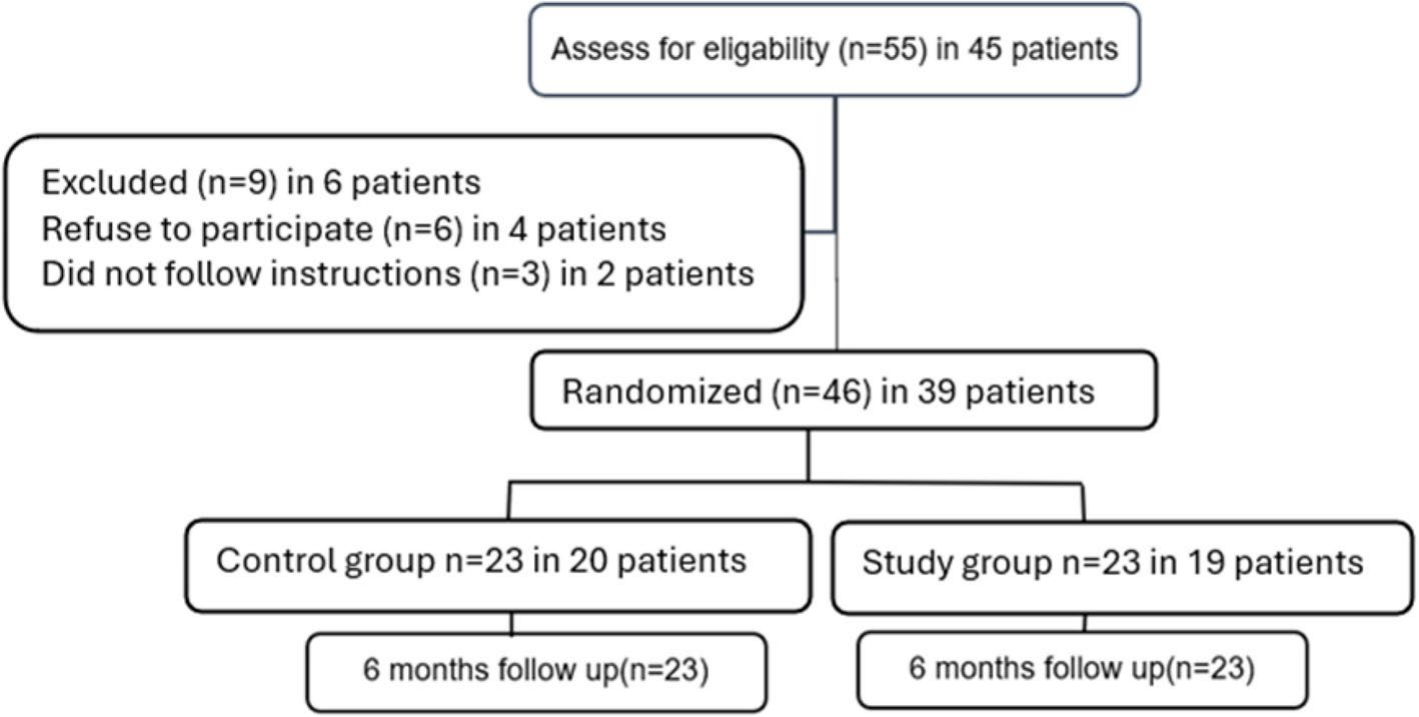
CONSORT flowchart

Participants were randomized in a 1:1 ratio to either the MINST or CNST group using a computer-generated random sequence prepared by an independent investigator who was not involved in patient recruitment, treatment, or outcome assessment. Randomization was performed at the patient level (cluster randomization); therefore, when a patient contributed more than one mandibular incisor, all eligible sites were allocated to the same treatment arm. Although randomization was performed at the patient level, the primary analysis was conducted at the site level, as clinical outcomes were assessed for individual teeth (Fig. 1). To account for the clustering of multiple sites within the same patient, all statistical analyses employed methods that adjust for within-patient correlation: generalized estimating equations (GEE) for binary outcomes and linear mixed-effects models with patient as a random intercept for continuous outcomes. Allocation concealment was achieved using sealed, opaque envelopes that were sequentially numbered, tamper-proof, securely stored, and opened only after baseline assessments were completed. Neither the recruiting clinician nor the treating periodontist had prior access to the randomization codes. Due to the nature of the interventions, operator blinding was not feasible. To reduce bias, all procedures were conducted under local anesthesia with similar treatment times, minimizing the chances of participants distinguishing between groups. While complete blinding of patients could not be fully achieved, this limitation is acknowledged. However, outcome examiners and radiographic assessors were completely blinded to group allocations. The radiographs were anonymized and coded, and the data were provided to the statistician using neutral group labels (A/B). To evaluate the effectiveness of blinding, outcome examiners were asked at the end of the study to guess the group assignments, and their responses did not significantly differ from random chance. The collected data were tested by using the exact binomial test (Table 1).

**Table 1:** Blinding assessment of outcome examiners

Examiner	Total Assessments (n)	Correctly Guessed (n)	Correct (%)	Expected Chance (%)	*p*-value
Examiner 1	46	21	45.7%	50%	0.63
Examiner 2	46	24	52.2%	50%	1.00
Overall	92	45	48.9%	50%	0.86

### Examiner calibration

Two examiners (S.H. and M.G.) were calibrated before the start of the study to ensure the reliability of probing depth (PPD) and clinical attachment level (CAL) measurements. Calibration was carried out on 10 non-study patients (40 sites). Each examiner recorded PPD and CAL twice, 48 h apart, using a UNC-15 probe. Intra-examiner and inter-examiner reliability were assessed using intra-class correlation coefficients (ICC) with 95% confidence intervals. Both examiners achieved excellent intra-examiner reliability (ICC for PPD = 0.94–0.96; ICC for CAL = 0.93–0.95) and inter-examiner agreement (ICC range 0.96–0.98). Weighted kappa for BOP exceeded 0.80, indicating substantial agreement. Periodic recalibration was conducted mid-study to avoid measurement drift.

### Clinical and radiographic examination

Two calibrated examiners recorded all clinical parameters; to ensure consistency, an inter-examiner calibration was conducted before data collection. The calibration involved measuring a subset of patients, with agreement assessed using intraclass correlation coefficients (ranging from 0.96 to 0.98) (S.H and M.G.). The primary outcome was pocket reduction, assessed as the change in probing pocket depth (PPD) from baseline to follow-up. Pocket closure (PC), defined as the proportion of sites achieving PPD ≤ 4 mm with no BOP, was evaluated as a secondary outcome derived from PPD and BOP measurements [14]. Probing pocket depth (PPD) was measured from the gingival margin to the base of the periodontal pocket, and gingival bleeding was assessed using a dichotomous full-mouth bleeding score (FMBS) [14]. Secondary outcomes also included clinical attachment level (CAL) measured from the cementoenamel junction (CEJ) to the base of the pocket [15].

Defect bone fill (DBF) was measured as the linear distance from the CEJ to the base of the intrabony defect (CEJ–BD) [16], and Full-mouth plaque score (FMPS) was recorded as previously described [17]. UNC-15 periodontal probe (Hu-Friedy, Chicago, Illinois, USA). At 6 sites, each tooth was used for pocket depth and CAL measurement. The deepest interproximal site was used as a test site. Tooth mobility was measured using Glickman’s index, with grade 1 indicating mobility slightly more than normal; grade 2 indicating mobility moderately more than normal; and grade 3 indicating severe mobility buccolingually and/or mesiodistally, combined with vertical displacement [3]. In addition to recording tooth mobility according to Miller’s classification, the level of bone support was evaluated radiographically and divided into three categories based on the extent of bone loss relative to root length (coronal third, middle third, and apical third). The distribution of mobility grades was subsequently analyzed in relation to these bone levels.

All teeth were evaluated for occlusal trauma, and occlusal adjustments were performed through selective grinding with fine burs on high-contact areas. If excessive forces were detected on the incisors due to lateral movement, the occlusion was adjusted to distribute these forces more evenly. The dental guidance mechanism was modified to ensure a better balance of force distribution. After making the necessary adjustments, a thorough occlusion reassessment was carried out using articulating paper to evaluate movement in all directions, including lateral and protrusive movements. Additionally, surface smoothing was performed following the reassessment to eliminate any high spots or irregularities that could lead to uneven force distribution. This systematic approach helped maintain optimal occlusal function and reduce the risk of dentoalveolar trauma. Patient satisfaction was also measured using the net promoter score (NPS), with promoters scoring 9–10, passives scoring 7–8, and detractors scoring 0–6. All clinical parameters were assessed at baseline, 3 months, and after 6 months of follow-up. This was done by a survey question: At the 6-month follow-up visit, each patient was asked the following question in a private, comfortable setting. The question was phrased in simple, clear Arabic to ensure patient comfort and understanding: *"On a scale of 0 to 10, how likely are you to recommend this treatment to a friend or colleague with a similar dental condition?"* Where: 0 = I would never recommend it, 1–4 = Very unlikely to recommend, 5–6 = Neutral, 7–8 = Likely to recommend, and 9–10 = I would strongly recommend it*.* Response Classification: Based on their responses, patients were categorized into three groups: Promoters: Score 9–10 (highly satisfied patients who are likely to recommend the treatment)*.* Passives: Score 7–8 (satisfied but not enthusiastic patients)*.* Detractors: Score 0–6 (dissatisfied patients who may spread negative feedback) [18]*.*

**Fig. 2:**
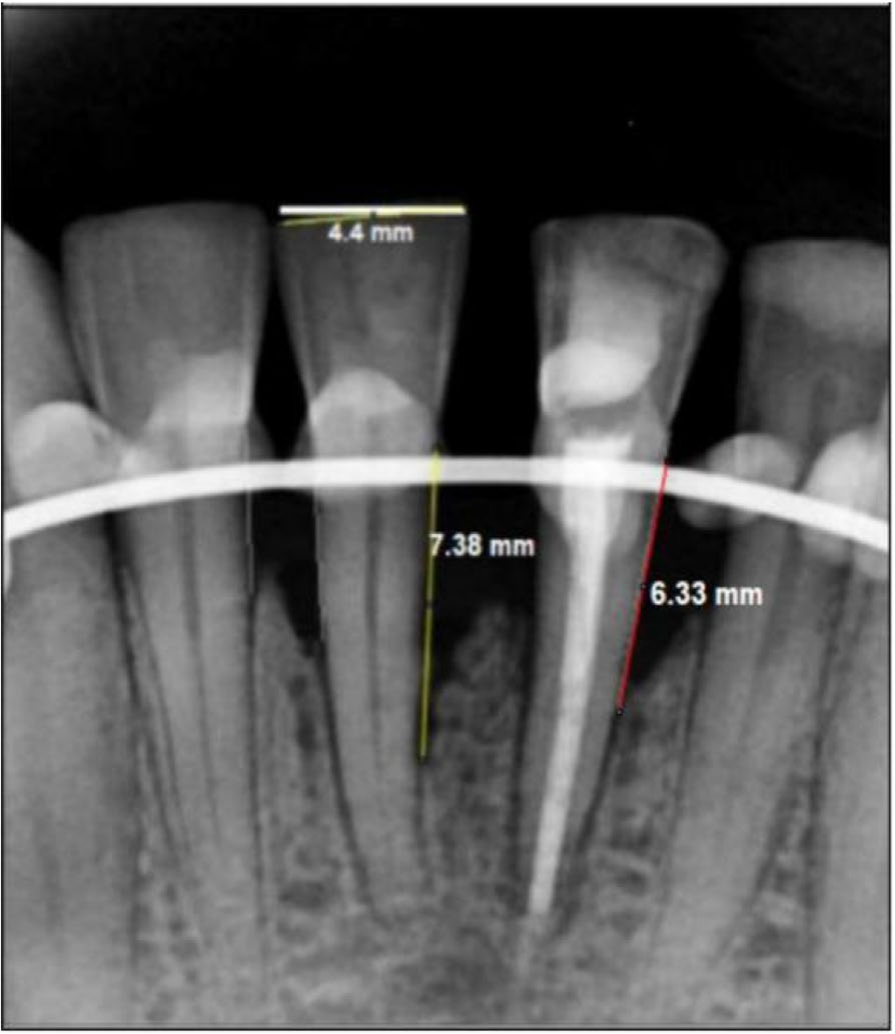
Perioperative radiographic measurements of a study MINST group

The long-cone paralleling technique was used for radiographic evaluation to obtain full-mouth periapical radiographs to identify bone destruction patterns and the amount of alveolar bone deficiency. Then, the sample periapical x-rays were taken at a 6-month follow-up. Image analysis was performed using ImageJ (National Institutes of Health, USA), an open-source image processing software. Radiographic images were taken by (manual Acteon PSPIX2, hypothetical goals 20Ip/mm. output time 1.6–1.7 s, measurements L154*D.204*H193mm. PSP IX2) acquired in JPG format and analyzed on a Windows 10 computer with a 1920 × 1080 resolution monitor [19]. A reference object with known dimensions was included in the image, as the tooth width from the most distal point to the most mesial point was used to calibrate peripheral radiography. A line was drawn along the reference object using ImageJ's "Straight Line Tool", and the actual length was input into the "Set Scale" function. Uniform scaling guarantee, millimeters (mm), was chosen as the measurement unit. Linear measurements were performed using the "Straight Line Tool" from CEJ to the crest of the bone (CB), measures obtained (Fig. 2).

### Intervention

All participants received step 1 periodontal therapy [20, 21], which included supragingival mechanical plaque removal to eliminate calculus and biofilm by ultrasonic scaler with conventional tips (Woodpecker K LED, China, Guilin, Guangxi Zhang Autonomous region). They also received motivation and proper oral hygiene instructions. Vitality tests were done for all lower incisor teeth using a combination of cold and electric pulp testing. All lower incisor teeth included in the study were lingually splinted permanently using Stainless steel orthodontic wire (0.5 mm–0.7 mm). The teeth were cleansed and isolated by a rubber dam. Etching gel (37% phosphoric acid) was applied to the enamel surface in a position where the wire would be adapted (for 30 s, then rinsed and dried). The wire was fabricated to extend one stable tooth from each side. The wire was passively adapted to avoid tension and discomfort for the patient. A bonding agent was applied over the etched enamel, then a flowable composite in small amounts over each tooth was applied, then a light-cure was used for at least 20 s, and finally the composite was polished with polishing discs [22, 23]. Non-vital teeth were root canal treated, followed by fabrication of a lingual splint. Four weeks after completion of step 1 therapy, all patients underwent subgingival instrumentation (step 2) under local anesthesia. In the CNST group, conventional subgingival instrumentation was performed by the same experienced periodontist (S.H.) without magnification. Debridement was initiated using an ultrasonic scaler (Woodpecker K LED, Guilin, China) equipped with standard tips, followed by hand instrumentation with a Columbia 13/14 universal curette (Hu-Friedy, Chicago, IL, USA) to ensure thorough root surface debridement. Continuous water cooling was maintained throughout the procedure. Each session lasted approximately 45 min and was completed when the root surface was judged smooth upon tactile evaluation. Final polishing was performed using a professional prophylaxis paste (Proxyt RDA 7, Ivoclar Vivadent, Schaan, Liechtenstein). In the MINST group, minimally invasive non-surgical therapy (MINST) protocol by Nibali (2019) [21, 24] was performed by the same operator under 4 × magnification loupes (Ergo Loupes, Guangdong, China). The procedure followed minimally invasive principles, emphasizing gentle and precise instrumentation to preserve soft tissue integrity. Fine ultrasonic tips were used in combination with mini-curettes (Gracey Mini-Five curettes, Hu-Friedy, Chicago, IL, USA) to allow controlled subgingival debridement with minimal tissue trauma. The duration of the session and final polishing procedure were identical to those of the CNST group, while local anesthesia was used without a vasoconstrictor, allowing better bleeding. A strict written protocol was followed in both groups to standardize the sequence, duration, and treatment endpoints. The same operator performed all procedures to eliminate operator-related variability. Baseline PPD, CAL, FMPS, and linear radiographic intrabony defect depth base fill (DBF) were assessed after treatment. All clinical parameters were reassessed at three and six months later. No further subgingival professional mechanical plaque removal was performed during the follow-ups. The use of a splint was reported to make patients' self-cleaning more challenging (Pasini et al., 2006) [22]. For this reason, meticulous proper Dental Health Education (DHE) sessions after splinting were held and reassured during follow-up periods [25]. This study adheres to CONSORT guidelines.

### Safety and adverse event monitoring

Throughout the study period, participants were monitored for any adverse events or complications related to the treatment procedures. Patients were instructed to report any unusual symptoms, including persistent pain, swelling, bleeding, infection, allergic reactions, or splint-related problems. In addition, patients were instructed to contact the investigator immediately in case of any post-operative symptoms. Adverse events were assessed and documented at each follow-up visit (3 and 6 months) using a standardized case report form. The treating periodontist evaluated the severity of each event (mild, moderate, or severe) based on clinical judgment, considering symptom intensity and the need for intervention.

### Statistical analysis

Data were collected and analyzed using IBM SPSS Statistics version 27 (IBM Corp., Armonk, NY, USA). Continuous variables, including probing pocket depth (PPD), clinical attachment level (CAL), and defect bone fill (DBF), were expressed in millimeters. Full-mouth plaque score (FMPS) and full-mouth bleeding score (FMBS) were expressed as mean scores, whereas patient satisfaction was expressed as percentages. The normality of continuous data was tested using the Shapiro–Wilk test. Descriptive statistics (means ± standard deviations) were calculated for PPD and CAL at baseline, 3 months, and 6 months, and for DBF at baseline and 6 months. Age was analyzed using one-way ANOVA. A linear mixed model (LMM) with restricted maximum likelihood estimation was employed to analyze PPD, CAL, and DBF, with group and time as fixed effects, and their interaction, and patient as a random intercept to account for clustering of multiple sites within the same patient. Pairwise comparisons were adjusted using the Bonferroni correction; in some intergroup comparisons, this adjustment resulted in adjusted p-values reported as p = 1.000, which reflects rounding after correction rather than an exact probability. The level of significance was set at p < 0.05. The Friedman test was used for intragroup comparisons of FMPS and FMBS as they were non-normally distributed, while the Kruskal–Wallis test was applied for intergroup comparisons. Patient satisfaction was analyzed using the Net Promoter Score (NPS), where patients were classified as promoters (score 9–10), passives (score 7–8), or detractors (score 0–6). The percentage of promoters and detractors was calculated. NPS was derived as (% Promoters – % Detractors), with a score NPS > 0: More Promoters than Detractors (generally positive), NPS = 0: Equal number of Promoters and Detractors, NPS < 0: More Detractors than Promoters (negative, requires attention), NPS ≥ 50: Excellent (world-class) patient satisfaction, NPS ≥ 70: Exceptional (top-tier) patient satisfaction. The proportion of sites achieving pocket closure (PPD ≤ 4 mm with no BOP) was calculated as percentages at 3 and 6 months.

## Results

### Blinding assessment of outcome examiners

The results of the blinding assessment indicate that both examiners were unable to correctly identify the treatment allocation beyond chance level. Examiner 1 correctly guessed the group assignment in 45.7% of cases (21/46), while Examiner 2 achieved 52.2% accuracy (24/46), with no statistically significant deviation from the expected 50% chance level (p = 0.63 and p = 1.00, respectively). When combining both examiners, the overall correct identification rate was 48.9% (45/92), which also did not differ significantly from chance (*p* = 0.86). These findings suggest that the outcome assessors were effectively blinded, as their ability to identify treatment groups was no better than random guessing Table 1.

### Demographic data

Table 2 presents the mean and standard deviation values for demographic data. The total sample size consisted of 46 sites from 39 patients, with 23 sites in each group. There were no significant differences in gender and age between the two groups (*p* > 0.05). In the CNST group, 20 patients exhibited 10 sites with grade II mobility and 13 sites with grade III mobility. In the MINST group, 19 patients had 11 sites with grade II mobility and 12 sites with grade III mobility, as shown in Table 3.

**Table 2:** Demographic characteristics of study groups

	CNST group	MINST group	P value
Gender (m/f)	9 (45%)/11 (55%)	9 (47.4%)/10 (52.6%)	0.88
Age	35.09 ± 8.56	34.39 ± 8.64	0.79

*CNST* Conventional non-surgical therapy (Control group), *MINST* Minimally invasive non-surgical therapy (Study group)

**Table 3:** Preoperative Grade of mobility and degree of bone loss

	Mobility									
	Grade 2					Grade 3				
	N	%	C1	M1	A1	N	%	C1	M1	A1
CNST group	10	43.5%	1	5	4	13	56.5%	-	7	6
MINST group	11	47.8%	-	5	6	12	52.2%	-	5	7
*P* value		0.77	0.28	0.83	0.51		0.77	-	0.54	0.54

C1 coronal one-third

M1 middle one-third

A1 apical one-third

### Clinical outcomes

For PPD, both groups demonstrated marked reductions over time. In the CNST group, mean PPD decreased from 7.91 ± 1.27 mm at baseline to 5.70 ± 0.97 mm at 3 months, and further to 4.17 ± 0.72 mm at 6 months (Δ = 3.74 mm, *p* < 0.001, intragroup). In the MINST group, PPD decreased from 8.74 ± 0.92 mm at baseline to 5.04 ± 1.55 mm in 3 months, and to 3.41 ± 1.40 mm at 6 months (Δ = 5.33 mm, *p* < 0.001, intragroup). Although the magnitude of reduction was numerically greater in the MINST group, the between-group comparison was not statistically significant (*p* = 1.000). For CAL, the CNST group improved from 8.39 ± 1.23 mm at baseline to 6.00 ± 1.41 mm in 3 months, and to 4.83 ± 1.07 mm at 6 months (Δ = 3.56 mm, p < 0.001, intragroup). MINST Group improved from 8.78 ± 1.13 mm at baseline to 5.91 ± 1.41 mm at 3 months, and to 4.13 ± 0.81 mm at 6 months (Δ = 4.65 mm, p < 0.001, intragroup). The time effect was highly significant (*p* < 0.001), confirming consistent clinical improvements across time points. However, no significant difference was detected between the two groups at any time point (*p* = 0.527, intergroup) (Table 4). Analysis of the pocket closure (PPD ≤ 4 mm with no BOP) revealed progressive improvement in both groups. In the CNST group, only 4.3% of sites achieved this endpoint at 3 months, increasing to 39.1% in 6 months. In the MINST group, the proportion improved from 30.4% to 65.2% over the same period. The intragroup comparison confirmed a significant improvement in both groups (p = 0.0098) for the CNST group and (*p* = 0.0377) for the MINST group, with a significantly higher overall success rate in the intergroup comparison (*p* = 0.015) (Table 5).

**Table 4:** PPD and CAL outcomes with intra- and intergroup comparisons

Outcome	Baseline (Mean ± SD)	3 Months (Mean ± SD)	6 Months (Mean ± SD)	Δ (0 → 3mo)	Δ (0 → 6mo)	Intragroup (*P* value)	Intergroup (*P* value)
PPD CNST	7.91 ± 1.28	5.70 ± 0.97	4.17 ± 0.72	2.22	3.74	*p* < 0.001	*p* = 0.934
PPD MINST	8.74 ± 0.92	5.04 ± 1.55	3.41 ± 1.40	3.70	5.33	*p* < 0.001	
CAL CNST	8.39 ± 1.23	6.00 ± 1.41	4.83 ± 1.07	2.39	3.57	*p* < 0.001	*p* = 0.424
CAL MINST	8.78 ± 1.13	5.91 ± 1.41	4.13 ± 0.81	2.87	4.65	*p* < 0.001

**Table 5:** Pocket closure N/% of sites with PPD ≤ 4 mm with no BOP, NPS, and Defect Bone Fill (DBF)

Group	Time	Total sites (n)	Sites with PPD ≤ 4 (n)	No-BOP (n)	No-BOP (% of total)	*P* value	*P*-value total
CNST	3	23	2	1	4.3%	0.0098*	0.015*
CNST	6	23	15	9	39.1%		
MINST	3	23	10	7	30.4%	0.0377*	
MINST	6	23	19	15	65.2%		
	Groups	Promoters%	Detractors%	Score	*P* value		
NPS	CNST	47%	9%	38	0.39		
	MINST	53%	11%	42			
	Group	Baseline (Mean ± SD)	6 months (Mean ± SD)	Δ (Change)	Intragroup (*P* value)	Intergroup (*P* value)	
DBF	CNST	8.03 ± 0.98	5.55 ± 1.17	2.48	0.001	0.973	
	MINST	8.22 ± 0.82	4.83 ± 1.13	3.39	0.001		

### Radiographic outcome

**Fig. 3:**
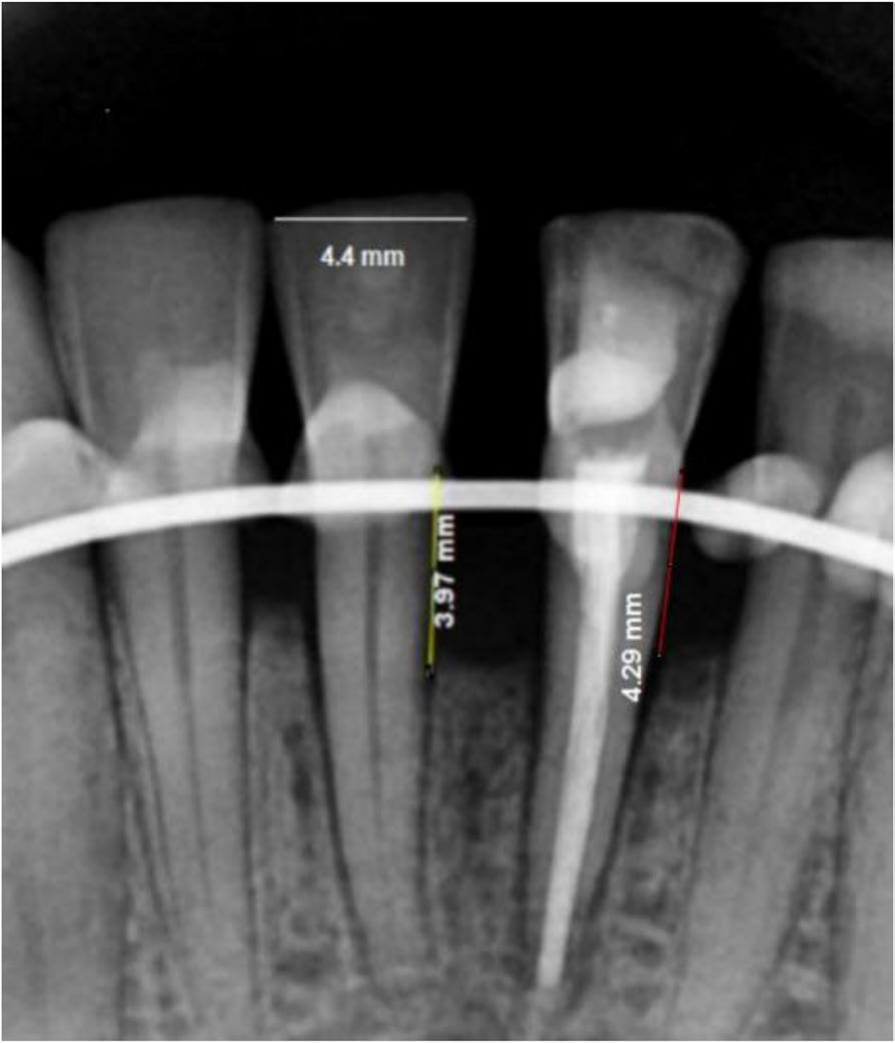
Post-operative radiographic measurements 6 months following therapy

Radiographic analysis confirmed a significant reduction in defect depth within both groups. In the CNST group, DBF decreased from 8.03 ± 0.98 mm at baseline to 5.55 ± 1.17 mm in 6 months (Δ = 2.48 mm, *p* = 0.001, intragroup). In the MINST group, DBF decreased from 8.22 ± 0.82 mm to 4.83 ± 1.13 mm (Δ = 3.39 mm, *p* = 0.001, intragroup). Although the reduction was numerically greater in the MINST group, the difference between groups was not statistically significant (*p* = 0.973, intergroup), Table 5, (Fig. 3).

### Patients’ satisfaction

Patient satisfaction levels were comparable between groups. CNST Group showed a promoter rate of 47% and a detractor rate of 9%, yielding an overall score of 38. MINST group showed slightly higher values with a promoter rate of 53% and a detractor rate of 11%, corresponding to a score of 42. However, this difference did not reach statistical significance (*p* = 0.39) (Table 5).

### FMBS and FMPS

A significant difference was reported in FMPS and FMBS in both groups after 6 months (*p* < 0.05) compared to baseline, with a reduction from 1.48 ± 0.51 to 0.65 ± 0.57 and from 1.57 ± 0.95 to 0.83 ± 0.49 in FMBS and FMPS, respectively, in the CNST group, while in the MINST group, FMBS reduced from 1.48 ± 0.51 to 0.83 ± 0.49 and FMPS from 1.65 ± 0.78 to 0.74 ± 0.54 with no significant difference between the 2 groups (Table 6).

**Table 6:** FMBS and FMPS

	Groups	Baseline mean ± SD	3 months mean ± SD	6 months mean ± SD	P value
FMBS	CNST group	1.48 ± 0.51	0.57 ± 0.51	0.65 ± 0.57	< 0.001a
	MINST group	1.48 ± 0.51	0.61 ± 0.5	0.69 ± 0.56	< 0.001a
	*P* value	1	0.77	0.78	
FMPS	CNST group	1.57 ± 0.95	0.65 ± 0.49	0.83 ± 0.49	< 0.00a
	MINST group	1.65 ± 0.78	0.61 ± 0.5	0.74 ± 0.54	< 0.00a
	*P* value	0.84	0.76	0.55	

^*^Significant difference between the control and the MINST group

^a^significant improvement between time points

### Adverse events

No treatment-related adverse events were reported in either group during the study period. Specifically, no cases of post-operative infection, allergic reactions, prolonged bleeding requiring intervention, or severe pain requiring additional medical attention were observed. Three cases of splint detachment were recorded (two in the MINST group and one in the CNST group), which were managed by re-cementation without further complications. No transient post-operative symptoms such as pain, sensitivity, or soft-tissue reactions were reported during the active follow-up period.

## Discussion

Patients with periodontitis often experience significant tooth mobility due to the loss of alveolar bone and attachment. Tooth splinting is commonly used in treatment, especially in the area of the mandibular anterior teeth. Mandibular incisors, in particular, are known for their greater mobility compared to other teeth in the dental arch. This increased mobility can be attributed to their unique anatomical structure, which consists of a single, conical root. The slender design of these roots creates a narrower surface area, limiting their ability to resist lateral forces. As a result, when subjected to occlusal or masticatory stress, mandibular incisors are more likely to move slightly within their alveolar sockets. This increased mobility becomes especially pronounced in cases of periodontal disease or occlusal trauma, where the supporting structures of the teeth may be compromised [26]. The front teeth of the mandible were also reported to show the most notable tooth loss and mobility in patients with stage III and IV periodontitis [27]. Patients with grade C periodontitis were excluded because of the rapid progression and poor predictability of treatment outcomes in this population. Including such cases could introduce high heterogeneity and potentially confound the effects of the evaluated interventions. Furthermore, current guidelines recommend more intensive or surgical approaches for grade C patients, which would not align with the non-surgical treatment protocols investigated in this trial [26].

Evidence demonstrates that during long-term supportive therapy, tooth mobility increases the chance of eventual attachment loss [10]. Furthermore, a clinical investigation has shown that regulating mobility in patients receiving therapy for periodontitis through occlusal adjustment may affect the clinical attachment level gained after treatment [28]. Splinting therapy lowers occlusal force and transfers forces from moving teeth to their immovable neighbors, but it can also impact interproximal dental hygiene practices [11, 29, 30]. Splinting is therefore frequently considered an adjunctive therapeutic approach to stabilize mobile teeth and improve patient comfort and function during periodontal treatment. By connecting mobile teeth to adjacent, more stable teeth, splints may help distribute occlusal forces more evenly and reduce functional discomfort. However, splinting does not address the underlying inflammatory etiology of periodontitis and should not be considered a substitute for causal periodontal therapy. Effective control of periodontal inflammation through appropriate non-surgical or surgical treatment remains essential for achieving long-term periodontal stability and preventing further attachment loss. Consequently, splinting is generally recommended only as a complementary measure within a comprehensive periodontal treatment plan rather than as a standalone intervention [31]. In contrast, opting for splinting without accompanying periodontal intervention often results in only temporary stability. Recently, MINST represents a shift in clinical practice towards less invasive, patient-centered care. Compared with CNST, there has been an emphasis on treating periodontitis with minimally invasive techniques to reduce patient discomfort and improve the likelihood of recovery [32, 33]. Therefore, the present study aimed to compare the effects of MINST and CNST, each with splinting, in treating stage III and IV periodontitis on treatment outcomes. During the procedure, anesthesia is used to allow for painless, optimal access into the full depth of the lesion [34].

In the present study, both treatment groups exhibited significant reductions in probing pocket depth (PPD) and gains in clinical attachment level (CAL) after three and six months, reflecting the biological response to biofilm disruption and bacterial load reduction following non-surgical therapy [8, 32]. Although the magnitude of change in PPD and CAL was comparable between groups, a significantly greater proportion of sites achieved pocket closure (PPD ≤ 4 mm without BOP) in the MINST group compared to the CNST group [32]. This advantage may be attributed to the use of mini-instruments and magnification in MINST, which minimizes tissue trauma, preserves soft tissues, and allows more precise subgingival debridement. These mechanisms are consistent with previous studies reporting enhanced clinical attachment gains and pocket closure following minimally invasive non-surgical approaches, sometimes avoiding the need for surgical intervention [34, 35].

In this study, both MINST and CNST demonstrated a significant increase in radiographic bone fill after six months of follow-up. The mean defect reduction was slightly greater in the MINST group; however, the intergroup comparison revealed no statistically significant difference. This suggests that both approaches were effective in promoting bone fill at sites with advanced mobility, where splinting was used to stabilize the teeth during treatment. The absence of a significant difference between groups may be related to the severity of initial periodontal destruction in splinted incisors with grade II–III mobility, which could limit the regenerative potential [36]. In addition, underlying biological mechanisms such as bone remodeling and defect morphology may have influenced the observed outcomes, although these parameters were not directly assessed in the present investigation [37].

Although pocket closure and improvements in clinical parameters (PPD and CAL) were significantly greater in the MINST group compared to CNST, no significant intergroup differences were observed in radiographic bone fill. This discrepancy may be explained by the fact that clinical healing, particularly pocket closure, is strongly influenced by soft tissue adaptation and reduced inflammation, which can occur independently of measurable radiographic bone changes in the short term. Radiographic assessment, on the other hand, is less sensitive to subtle remodeling and may require longer follow-up to detect meaningful differences between groups. Furthermore, the presence of splinted mandibular incisors with advanced mobility could have contributed to a slower rate of bone remodeling despite the clinical stability achieved. Previous reports also highlight that radiographic bone fill often lags behind clinical attachment gains, and true regeneration cannot be fully confirmed without histological analysis. Furthermore, current literature indicates that most studies comparing MINST and CNST focus on clinical parameters (PPD and CAL), while robust data on intergroup differences in radiographic bone fill remain limited.

In the present study, patient satisfaction scores did not differ significantly between the MINST and CNST groups, as reflected by comparable Net Promoter Scores (38 vs. 42, p = 0.39). This similarity may be explained by the splinting procedure itself, which reduced tooth mobility and alleviated preoperative discomfort in both groups, thereby enhancing patients’ perception of treatment success [32]. Additional factors, including occlusal adjustment and the use of local anesthesia, likely contributed to equalizing the overall patient experience. Although previous studies have suggested that minimally invasive techniques are associated with reduced intraoperative trauma and greater comfort, the relief from mobility achieved by splinting may have outweighed these differences in the current population. Nevertheless, this interpretation remains tentative, as determinants of satisfaction were not systematically assessed [11, 38].

### Limitations

This study has several limitations. First, the 6-month follow-up period is relatively short and may not adequately reflect the long-term stability of splinted mandibular incisors, particularly after splint removal. Second, the study focused solely on mandibular incisors, which may limit the generalizability of the findings to other tooth types and anatomical sites. Third, radiographic bone fill was assessed using two-dimensional radiographs, which may not accurately capture three-dimensional changes. Fourth, the morphology of intrabony defects (e.g., number of remaining osseous walls) was not classified, although this factor may significantly influence outcomes. Fifth, patient satisfaction was measured only through the Net Promoter Score, without a comprehensive analysis of pain, function, or esthetics. Sixth, the relatively small sample size may have reduced the statistical power to detect subtle differences. Seventh, the same operator performed both interventions, which could introduce operator bias. Finally, the Hawthorne effect cannot be excluded, as patients may have improved their oral hygiene behavior simply due to being observed in a clinical trial setting. Although protocol modifications were made before data collection, the ClinicalTrials.gov registry was updated after study completion, which is acknowledged as a limitation in reporting transparency. Specifically, the eligibility criteria applied in this study differed from those originally registered in ClinicalTrials.gov. These differences included the age range (18–55 vs. 18–60 years), periodontal mobility grade inclusion criteria (Grade 2 vs. Grade 2–3), and smoking status criteria (complete exclusion of smokers vs. exclusion of individuals smoking more than 10 cigarettes per day). These discrepancies should be considered when interpreting the findings and may limit the generalizability of the results to broader patient populations.

## Conclusion

Within the limitations of this study, both MINST and conventional non-surgical therapy (CNST) combined with splinting were effective in improving clinical and radiographic outcomes in stage III–IV periodontitis affecting mobile mandibular incisors. Both groups demonstrated significant reductions in probing pocket depth (PPD) and gains in clinical attachment level (CAL). Although a higher proportion of sites in the MINST group achieved pocket closure, no significant differences were observed between the groups for PPD, CAL, or radiographic bone fill. Therefore, the potential advantage of MINST should be interpreted cautiously. Patient-reported outcomes were similar in both groups, suggesting that both approaches are clinically effective and well-tolerated.

## Data Availability

All data or materials generated or analyzed during this study are included in this article.

## Abbreviations

ANOVA: Analysis of Variance
BD: Base of Defect
BMC: BioMed Central
BOP: Bleeding on Probing
CAL: Clinical Attachment Level
CB: Crest of Bone
CEJ: Cementoenamel Junction
CNST: Conventional Non-Surgical Periodontal Therapy
CONSORT: Consolidated Standards of Reporting Trials
DBF: Defect Bone Fill
DHE: Dental Health Education
FMBS: Full-Mouth Bleeding Score
FMPS: Full-Mouth Plaque Score
ICC: Intraclass Correlation Coefficient
LMM: Linear Mixed Model
MINST: Minimally Invasive Non-Surgical Therapy
Mm: Millimeter
NPS: Net Promoter Score
PPD: Probing Pocket Depth
PSP: Photostimulable Phosphor Plate
RCT: Randomized Controlled Trial
SEM: Scanning Electron Microscopy
SPSS: Statistical Package for the Social Sciences
UNC: University of North Carolina (periodontal probe)

## References

[1] Könönen E, Gursoy M, Gursoy UK. Periodontitis: a multifaceted disease of tooth-supporting tissues. J Clin Med. 2019. 10.3390/jcm8081135. 31370168 10.3390/jcm8081135 PMC6723779

[2] Dommisch H, et al. Efficacy of tooth splinting and occlusal adjustment in patients with periodontitis exhibiting masticatory dysfunction: a systematic review. J Clin Periodontol. 2022;49(Suppl 24):149–66. 10.1111/jcpe.13563. Epub 2021 Dec 1. 34854115 10.1111/jcpe.13563

[3] Kim GY, Kim S, Chang JS, Pyo SW. Advancements in methods of classification and measurement used to assess tooth mobility: a narrative review. J Clin Med. 2023;13(1):142. 10.3390/jcm13010142. 38202149 10.3390/jcm13010142 PMC10779763

[4] Citterio F, et al. Pocket closure and residual pockets after non-surgical periodontal therapy: a systematic review and meta-analysis. J Clin Periodontol. 2022;49(1):2–14. 10.1111/jcpe.13547. 34517433 10.1111/jcpe.13547 PMC9298904

[5] Graziani F, Karapetsa D, Alonso B, Herrera D. Nonsurgical and surgical treatment of periodontitis: how many options for one disease? Periodontol 2000. 2017;75(1):152–88. 10.1111/prd.12201. 28758300 10.1111/prd.12201

[6] Nascimento GG, Leite FRM, Pennisi PRC, López R, Paranhos LR. Use of air polishing for supra- and subgingival biofilm removal for treatment of residual periodontal pockets and supportive periodontal care: a systematic review. Clin Oral Investig. 2021;25(3):779–95. 10.1007/s00784-020-03762-y. 33464417 10.1007/s00784-020-03762-y

[7] Rateitschak-Plüss EM, Schwarz JP, Guggenheim R, Düggelin M, Rateitschak KH. Non-surgical periodontal treatment: where are the limits? An SEM study. J Clin Periodontol. 1992;19(4):240–4. 10.1111/j.1600-051x.1992.tb00460.x. 1373744 10.1111/j.1600-051x.1992.tb00460.x

[8] Kučič AC, Gašperšič R. Minimally invasive non-surgical therapy (MINST) in stage III periodontitis patients: 6-month results of a split-mouth, randomised controlled clinical trial. Clin Oral Investig. 2023;27(5):2075–87. 10.1007/s00784-023-04994-4. 37014505 10.1007/s00784-023-04994-4 PMC10071470

[9] Cobb CM, Sottosanti JS. A re-evaluation of scaling and root planing. J Periodontol. 2021;92(10):1370–8. 10.1002/JPER.20-0839. 33660307 10.1002/JPER.20-0839

[10] Hui S, Hong Y, Chen S, Bao Y, Song J, Zhao Y, et al. Efficacy of maxillary occlusal splints on mobile posterior teeth in patients with stage III or IV periodontitis during steps 1 and 2 of periodontal therapy: a randomized parallel-controlled clinical trial. Clin Oral Invest. 2025;29:425. 10.1007/s00784-025-06506-y 40875065

[11] Zhang Y, et al. Survival of nonsurgically splinted mandibular anterior teeth during supportive maintenance care in periodontitis patients. J Dent Sci. 2023;18(1):229–36. 10.1016/j.jds.2022.05.025. 36643235 10.1016/j.jds.2022.05.025 PMC9831790

[12] Tonetti MS, Greenwell H, Kornman KS. Staging and grading of periodontitis: framework and proposal of a new classification and case definition. J Clin Periodontol. 2018;45(January):S149–61. 10.1111/jcpe.12945. 29926495 10.1111/jcpe.12945

[13] Abramson JH. The Cornell Medical Index , a health questionnaire , has possibilities as an epidemiologic tool . The author discusses the uses of the index , as well as its limitations , and suggests modifications to enhance its usefulness . The Cornell Medical Index as. Am J Public Health. 1966;56(2):287–98. 10.2105/ajph.56.2.287 PMC1256865 5948222

[14] Trombelli L, Farina R, Vecchiatini R, Maietti E, Simonelli A. A simplified composite outcome measure to assess the effect of periodontal regenerative treatment in intraosseous defects. J Periodontol. 2020;91(6):723–31. 10.1002/JPER.19-0127. 31755986 10.1002/JPER.19-0127

[15] Karayiannis A, Lang NP, Joss A, Nyman S. Bleeding on probing as it relates to probing pressure and gingival health in patients with a reduced but healthy periodontium: a clinical study. J Clin Periodontol. 1992;19(7):471–5. 10.1111/j.1600-051X.1992.tb01159.x. 1430282 10.1111/j.1600-051x.1992.tb01159.x

[16] Abd El-Azeem SH, Khalil AA, Ibrahim MAM, Gamal AY. The use of integrin binding domain loaded hydrogel (RGD) with minimally invasive surgical technique in treatment of periodontal intrabony defect: a randomized clinical and biochemical study. J Appl Oral Sci. 2023;31:1–10. 10.1590/1678-7757-2023-0263. 10.1590/1678-7757-2023-0263 PMC10786453 38126565

[17] Patrick H, et al. Detection of periodontal bone loss on periapical radiographs—a diagnostic study using different convolutional neural networks. J Clin Med. 2023;12:7189. 38002799 10.3390/jcm12227189 PMC10672399

[18] Dawes JG. “The net promoter score: what should managers know?,.” Int J Mark Res. 2024;66(2–3):182–98. 10.1177/14707853231195003.

[19] Ferreira T, Rasband W. ImageJ User Guide User Guide ImageJ. Image J user Guide. 2012;1.46r. 10.1038/nmeth.2019.

[20] Sanz M, et al. “Treatment of stage I–III periodontitis—the EFP S3 level clinical practice guideline,.” J Clin Periodontol. 2020;47(S22):4–60. 10.1111/jcpe.13290. 32383274 10.1111/jcpe.13290 PMC7891343

[21] Kahler B, Hu JY, Marriot-Smith CS, Heithersay GS. Splinting of teeth following trauma: a review and a new splinting recommendation. Aust Dent J. 2016;61:59–73. 10.1111/adj.12398. 26923448 10.1111/adj.12398

[22] Pasini S, Bardellini E, Casula I, Flocchini P, Majorana A. “Effectiveness of oral hygiene protocol in patients with post-traumatic splinting.,.” Eur J Paediatr Dent. 2006;7(1):35–8. 16646643

[23] Thomson WM, Hashim R, Pack ARC. “The prevalence and intraoral distribution of periodontal attachment loss in a birth cohort of 26‐year‐olds,.” J Periodontol. 2000;71(12):1840–5. 10.1902/jop.2000.71.12.1840. 11156040 10.1902/jop.2000.71.12.1840

[24] Nibali L, Koidou V, Salomone S, et al. Minimally invasive non-surgical vs. surgical approach for periodontal intrabony defects: a randomised controlled trial. Trials. 2019;20:461. 10.1186/s13063-019-3544-8. 31351492 10.1186/s13063-019-3544-8 PMC6660941

[25] Doshi Y, Mani A, Marawar P, Mishra P. “A clinical study on mobility of teeth as assessed through their damping characteristics and progress of periodontal disease using advanced diagnostic aids: Mobilometer and Florida probe,.” J Int Clin Dent Res Organ. 2010;2(1):12. 10.4103/2231-0754.89987.

[26] Kwon TH, Lamster IB, Levin L. “Current concepts in the management of periodontitis,.” Int Dent J. 2021;71(6):462–76. 10.1111/idj.12630. 34839889 10.1111/idj.12630 PMC9275292

[27] Sulijaya B, Hutomo DI, Jesson A, Rahdewati H, Tadjoedin FM. Periodontal status in periodontitis patients with temporary periodontal splint: a retrospective study. Open Dent J. 2024;18(1):1–9. 10.2174/0118742106313216240620110123.

[28] Burgett FG, Ramfjord SP, Nissle RR, Morrison EC, Charbeneau TD, Caffesse RG. A randomized trial of occlusal adjustment in the treatment of periodontitis patients. J Clin Periodontol. 1992;19(6):381–7. 10.1111/j.1600-051x.1992.tb00666.x. PMID: 1634627. 1634627 10.1111/j.1600-051x.1992.tb00666.x

[29] Gkantidis N, Christou P, Topouzelis N. The orthodontic-periodontic interrelationship in integrated treatment challenges: a systematic review. J Oral Rehabil. 2010;37(5):377–90. 10.1111/j.1365-2842.2010.02068.x. 20202098 10.1111/j.1365-2842.2010.02068.x

[30] Graetz C, Ostermann F, Woeste S, Sälzer S, Dörfer CE, Schwendicke F. Long-term survival and maintenance efforts of splinted teeth in periodontitis patients. J Dent. 2019;80:49–54. 10.1016/j.jdent.2018.10.009. 30389428 10.1016/j.jdent.2018.10.009

[31] Herrera D, Sanz M, Kebschull M, Jepsen S, Sculean A, Berglundh T, et al. Treatment of stage IV periodontitis: the EFP S3 level clinical practice guideline. J Clin Periodontol. 2022;49(Suppl 24):4–71. 10.1111/jcpe.13639. PMID: 35688447. 35688447 10.1111/jcpe.13639

[32] Chung WC, Huang CF, Feng SW. Clinical benefits of minimally invasive non-surgical periodontal therapy as an alternative of conventional non-surgical periodontal therapy—A pilot study. Int J Environ Res Public Health. 2022. 10.3390/ijerph19127456. 35742702 10.3390/ijerph19127456 PMC9223734

[33] Nibali L, Pometti D, Chen TT, Tu YK. “Minimally invasive non-surgical approach for the treatment of periodontal intrabony defects: a retrospective analysis,.” J Clin Periodontol. 2015;42(9):853–9. 10.1111/jcpe.12443. 26257238 10.1111/jcpe.12443

[34] Lessang R, Purba MR, Ayuningtyas D. Efficacy of minimally invasive non-surgical therapy (MINST) in managing stage III/IV periodontitis: a systematic review of randomized controlled trials. Swiss Dent J. 2025;135(2):72–85. 10.61872/sdj-2025-02-08. 40697109 10.61872/sdj-2025-02-08

[35] Iorio-Siciliano V, Blasi A, Mauriello L, Salvi GE, Ramaglia L, Sculean A. Non-surgical treatment of moderate periodontal intrabony defects with adjunctive cross-linked hyaluronic acid: a single-blinded randomized controlled clinical trial. J Clin Periodontol. 2025;52(2):310–22. 10.1111/jcpe.14078. 39402910 10.1111/jcpe.14078 PMC11743238

[36] Mehta J, et al. “Minimally invasive non-surgical periodontal therapy of intrabony defects: a prospective multi-centre cohort study,.” J Clin Periodontol. 2024;51(7):905–14. 10.1111/jcpe.13984. 38710583 10.1111/jcpe.13984

[37] Cirelli JA, Fiorini T, Moreira CHC, de Molon RS, Dutra TP, Sallum EA. “Periodontal regeneration: is it still a goal in clinical periodontology?,.” Braz Oral Res. 2021;35(Supplement 2):1–15. 10.1590/1807-3107bor-2021.vol35.0097. 10.1590/1807-3107bor-2021.vol35.0097 34586211

[38] Sculean A, et al. A paradigm shift in mechanical biofilm management? Subgingival air polishing: a new way to improve mechanical biofilm management in the dental practice. Quintessence Int. 2013;44(7):475–7. 23616981 10.3290/j.qi.a29615

